# A New Fitting Method for Ambipolar Diffusion Length Extraction in Thin Film Structures Using Photoluminescence Measurement with Scanning Excitation

**DOI:** 10.1038/s41598-020-62093-w

**Published:** 2020-03-23

**Authors:** Cheng-Hao Chu, Ming-Hua Mao, You-Ru Lin, Hao-Hsiung Lin

**Affiliations:** 1 Graduate Institute of Electronics Engineering, National Taiwan University, No. 1, Roosevelt Rd. Sec. 4, Taipei, 10617 Taiwan; 2 Department of Electrical Engineering, National Taiwan University, No. 1, Roosevelt Rd. Sec. 4, Taipei, 10617 Taiwan; 3 Graduate Institute of Photonics and Optoelectronics, National Taiwan University, No. 1, Roosevelt Rd. Sec. 4, Taipei, 10617 Taiwan

**Keywords:** Optical physics, Optical techniques

## Abstract

A new simple method is proposed to extract the ambipolar diffusion length for two-dimensional (2D) electronic transport in thin film structures using a scanning photoluminescence microscopy (SPLM) setup. No spatially-resolved photoluminescence detection methods are required. By measuring the excitation-position-dependent PL intensity across the edge of a semiconductor, ambipolar diffusion length can be extracted from the SPLM profile through a simple analytic fitting function. Numerical simulation was first used to verify the fitting method. Then the fitting method was applied to extract the ambipolar diffusion length from the measured SPLM profile of a GaAs thin film structure. Carrier lifetime was obtained in an accompanying time-resolved photoluminescence measurement under the same excitation condition, and thus the ambipolar diffusion coefficient can be determined simultaneously. The new fitting method provides a simple way to evaluate carrier transport properties in 2D electronic transport structures such as thin films or quantum wells.

## Introduction

Carrier transport in semiconductors plays an essential role in the optimization of semiconductor optoelectronic devices such as lasers, light-emitting diodes (LEDs), and photodetectors. Particularly, carrier transport in an epitaxially-grown heterostructure will dramatically influence its optical and electrical properties. Studies on the carrier diffusion length and carrier diffusion coefficient provide the figure of merit for optoelectronics. In the literature, several reported techniques for determining the carrier diffusion length or diffusion coefficient in semiconductor thin film structures were based on current-voltage or light-current measurement^1–3^, photoluminescence (PL) measurement^4–9^, cathodoluminescence imaging^10–12^, or ultrafast measurement^13–18^, surface photovoltage measurement^19,20^. Among these techniques, the PL measurement with scanning excitation^7^ has the advantages of simple optical setup, easy sample preparation, relatively strong collected signal, and no need to sacrifice spatial resolution for better signal-to-noise ratio. However, no direct extraction is available due to the lack of a simple analytic fitting function. Therefore, numerical simulations must be performed to observe different carrier distribution profiles with different carrier diffusion lengths and then choose one to fit the experimental results through comparison^7^. In this study, we developed a compact fitting method to extract the ambipolar diffusion length using a micro-PL measurement setup with a scanning excitation source. This technique can be called scanning photoluminescence microscopy (SPLM). The concept of SPLM is similar to scanning photocurrent microscopy (SPCM) and the electron beam induced current (EBIC) technique, which are extensively applied to explore carrier transport properties in semiconductor nanowires^21,22^ and 2D materials^23,24^. Carrier lifetime under the same excitation condition was also obtained in an accompanying time-resolved photoluminescence (TRPL) measurement, and thus the ambipolar diffusion coefficient can be determined simultaneously. The fitting method was first verified by numerical simulation and then demonstrated to analyze the measured SPLM profile of a GaAs thin film structure. Ambipolar diffusion length, carrier lifetime, and ambipolar diffusion coefficient were obtained. The fitting method also can be applied to study 2D carrier transport in quantum wells.

## Results and Discussion

**Figure 1:**
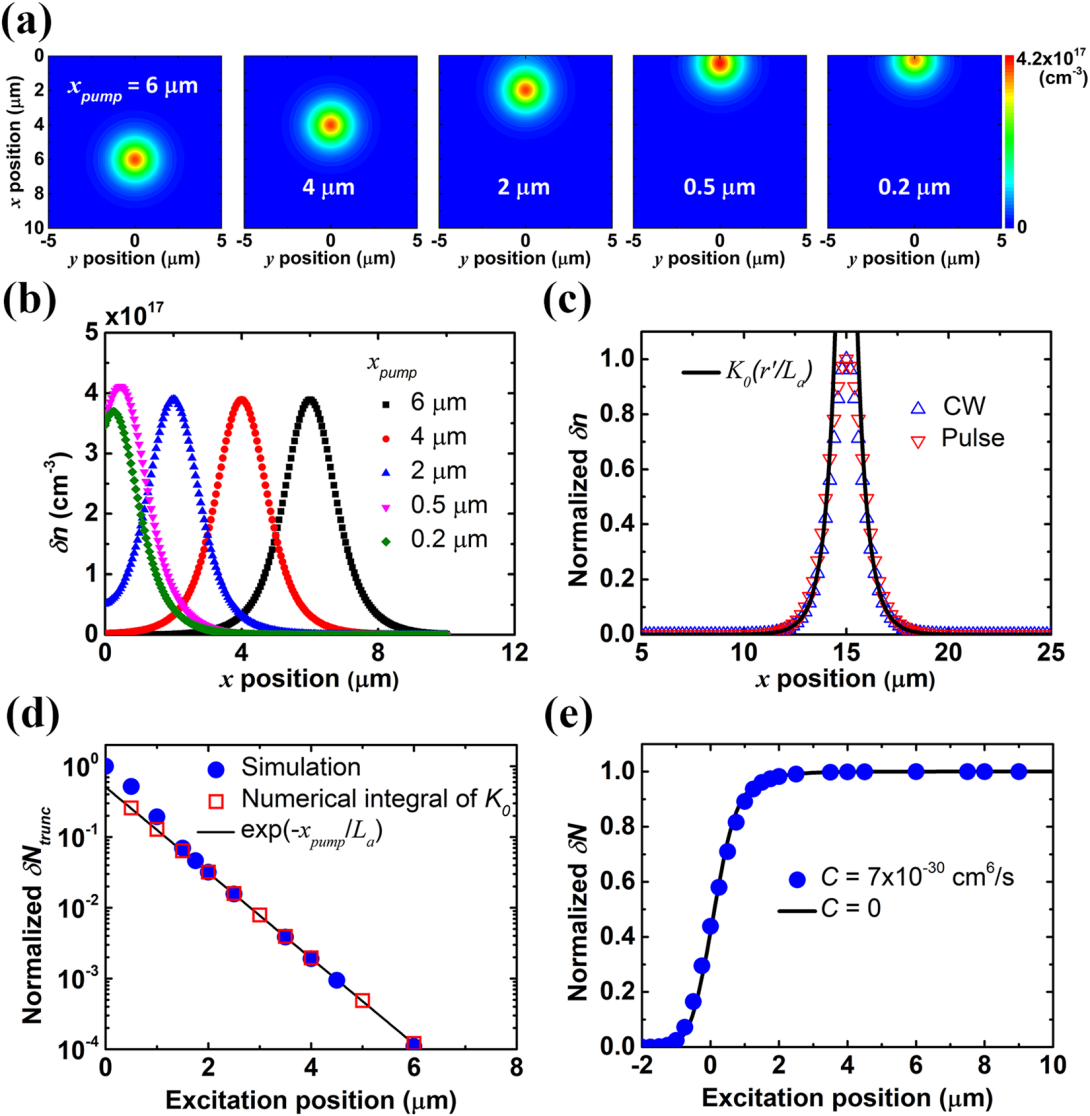
Simulation results for 2D ambipolar diffusion under CW or pulse excitation. (**a**) 2D photocarrier distribution mappings under CW excitation with varied excitation position (*x*, *y*) = (*x*_*pump*_, 0). (**b**) Cross-sections of the above mappings at *y* = 0 (**c**) Normalized photocarrier distribution under CW excitation and time-integrated photocarrier distribution under pulse excitation. Excitation position is 15 μm. The analytic function ${K}_{0}(r\text{'}/{L}_{a})$ is also shown for comparison. (**d**) Normalized truncated photocarrier number *δN*_*trunc*_ under pulse excitation as a function of excitation position *x*_*pump*_. The values calculated by the numerical integral of ${K}_{0}(r\text{'}/{L}_{a})$ and by the exponential function $\exp (-{x}_{pump}/{L}_{a})$ are also shown in the figure. (**e**) Scanning profile for the normalized total photocarrier number *δN* under pulse excitation. The result without Auger recombination is also shown for comparison. The excitation is scanned along *y* = 0 line for (**a**-**e**). Ambipolar diffusion length *L*_*a*_ is 720 nm, and surface recombination velocity of the GaAs/air interface at *x* = 0 boundary is 10^5^ cm/s. Excitation spot size and pulse width are 1 μm and 130 fs, respectively. The data points in (**b**,**c**) are sampled for clarity.

Numerical simulation for 2D ambipolar diffusion is performed to investigate the carrier transport in SPLM measurement. Ambipolar diffusion in a semi-infinite GaAs plane at position *x* > 0 is considered. The ambipolar diffusion equation is given by^25^

1 $$\frac{\partial \delta n(x,y,t)}{\partial t}={D}_{a}{\nabla }^{2}\delta n(x,y,t)+G(x,y,t)-R(x,y,t),$$

where *x* and *y* are spatial coordinates, and *t* is time. *x* = 0 corresponds to the GaAs sample boundary. *δn* is the photocarrier concentration in the transport plane, *D*_*a*_ is the ambipolar diffusion coefficient, *G* is the generation rate of optical excitation, and *R* is the recombination rate. Figure 1 shows the simulated results for 2D ambipolar diffusion when a continuous-wave (CW) or pulse excitation is incident near the *x* = 0 boundary of a semi-infinite GaAs plane. The excitation profile *G* is set to be a Gaussian function centered at excitation position (*x*, *y*) = (*x*_*pump*_, 0) with spot size of 1 μm for numerical simulation. Pumping density is about 290 kW/cm^2^, *D*_*a*_ is 20.4 cm^2^/s from the SPLM and TRPL measurements below, and surface recombination velocity at *x* = 0 boundary is 10^5^ cm/s^26^. Recombination with constant carrier lifetime *τ* of 254 ps from TRPL measurements and Auger recombination with Auger coefficient^27^ *C* of 7 × 10^−30^ cm^6^/s are generally used when calculating the recombination rate unless otherwise specified. Ambipolar diffusion length *L*_*a*_ of 720 nm can be obtained using the equation ${L}_{a}=\sqrt{{D}_{a}\tau }$. 2D photocarrier distribution mappings under CW excitation and their cross-sections at *y* = 0 are shown in Fig. 1a,b. It is found that the photocarrier distributions in Fig. 1b remain almost the same except being truncated at the boundary. When the excitation position approaches the *x* = 0 recombination boundary and the distance between the excitation positon and the boundary is smaller than *L*_*a*_, the amplitude of the photocarrier distribution begins to change due to the vicinity of the boundary. Therefore, for *x*_*pump*_ not in the vicinity of the boundary, the total photocarrier number *δN* with a given excitation position *x*_*pump*_ can be approximated as the total photocarrier number in an infinite plane minus the truncated photocarrier number which can be calculated from the integral of the ideal photocarrier distribution in an infinite plane *δn*_*inf*_ in the truncated source free region. The expression of *δN* may be written as

2 $$\delta N({x}_{pump})\approx \delta {N}_{inf}-\delta {N}_{trunc}({x}_{pump})$$

where *δN*_*inf*_ is the total photocarrier number in an infinite plane and *δN*_*trunc*_ is the truncated photocarrier number for excitation at *x* = *x*_*pump*_. Details of the deduction is shown in the Supplementary Information. Normalized photocarrier distribution under CW excitation and time-integrated photocarrier distribution under pulse excitation are depicted in Fig. 1c. Excitation position of 15 μm is chosen to avoid being in vicinity of the boundary. In order to solve Eq. (1) analytically, generation rate *G* is assumed to be a delta function centered at (*x*, *y*) = (*x*_*pump*_, 0). In the source free region where (*x*, *y*) ≠ (*x*_*pump*_, 0), with constant lifetime independent of excess carrier density, Eq. (1) in the steady state and in the polar coordinate system will become the Bessel equation. Therefore, its solution will have the form $\delta n(r\text{'}) \sim {K}_{0}(r\text{'}/{L}_{a})$ in an infinite plane, where $r{\prime} =\sqrt{{(x-{x}_{pump})}^{2}+{y}^{2}}$, *L*_*a*_ is the ambipolar diffusion length, and *K*_0_ is the modified Bessel function of the second kind^28^. The analytic function ${K}_{0}(r{\prime} /{L}_{a})$ is shown in Fig. 1c for comparison. The excitation profile *G* for pulse excitation is set to be a Gaussian function in both space and time with spot size of 1 μm, pulse duration of 130 fs, and repetition rate of 76 MHz for numerical simulation. Pumping density is about 290 kW/cm^2^. For a linear time-invariant system, the unit step response can be expressed as the convolution of impulse response and the unit step function^29,30^. Therefore, the time-integrated photocarrier distribution under pulse excitation has the form of the steady state analytic solution ${K}_{0}(r\text{'}/{L}_{a})$. Details of the derivation is presented in the Supplementary Information. Please note that carrier lifetime *τ* of 254 ps from TRPL measurements is much shorter than the pulse excitation period 13.2 ns and the unit step response will reach the steady state without any problem. However, Auger recombination may not be negligible under pulse excitation due to instantaneous high pumping density, and therefore Eq. (1) may become nonlinear. Thus, the results with Auger recombination need to be verified using numerical simulation. It is found that normalized time-integrated photocarrier distribution under pulse excitation with Auger coefficient *C* of 7 × 10^−30^ cm^6^/s is almost the same as that with *C* = 0, and both results show great consistency with the photocarrier distribution under CW excitation and the analytic function outside the source region. Thus, for excitation position *x*_*pump*_ outside the vicinity of the boundary, *δN*_*trunc*_ as a function of excitation position can be further written as

3 $$\delta {N}_{trunc}({x}_{pump})={N}_{0}{\int }_{-{\rm{\infty }}}^{{\rm{\infty }}}{\int }_{-{\rm{\infty }}}^{0}{K}_{0}(r{\prime} /{L}_{a})dx\,dy$$

where *N*_0_ is a factor for photocarrier number. The simulation results for the normalized truncated photocarrier number *δN*_*trunc*_ under pulse excitation as a function of excitation position are shown in Fig. 1d. The excitation is scanned along *y* = 0 line. The values calculated by the numerical integral of ${K}_{0}(r\text{'}/{L}_{a})$ are also shown in the figure. As expected, the profile of *δN*_*trunc*_ coincides with the numerical integral of ${K}_{0}(r\text{'}/{L}_{a})$ when *x*_*pump*_ is not in the vicinity of the boundary. Surprisingly, the numerical integral of ${K}_{0}(r\text{'}/{L}_{a})$ as a function of *x*_*pump*_ resembles the exponential function $\exp (-{x}_{pump}/{L}_{a})$. Thus the total photocarrier number *δN* exhibits the functional form

4 $$\delta N({x}_{pump})=a-b\,{\rm{\exp }}(-{x}_{pump}/{L}_{a})$$

where *a* and *b* are the parameters to be determined. Therefore, the key parameter *L*_*a*_, the ambipolar diffusion length, can be extracted by subtracting the profile of *δN* from its peak value and performing linear fitting in logarithmic scale. The simulated scanning profile for the normalized *δN* under pulse excitation in the transport plane is shown in Fig. 1e. The results without Auger recombination are also shown in the figure, and it is found that the influence of Auger recombination on the scanning profile is insignificant under such excitation condition. Note that the approximation in Eqs. (2,3) may not hold when the excitation position is in the vicinity of the boundary. Thus, that region should be excluded when one fits the profile of *δN*.

**Figure 2:**
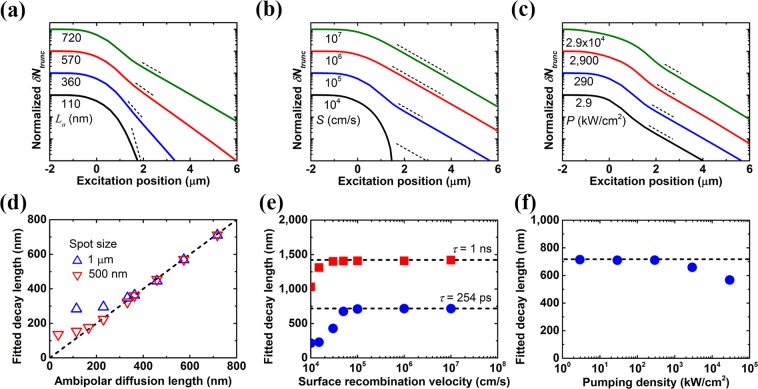
Simulation results for the truncated photocarrier number *δN*_*trunc*_ and the fitted decay length *L*_*fit*_ under pulse excitation. (**a**-**c**) Profiles of *δN*_*trunc*_ with varied parameters such as (**a**) ambipolar diffusion length *L*_*a*_, (**b**) surface recombination velocity of the GaAs/air interface *S*, and (**c**) pumping density *P*, respectively. (**d**) Relation between the fitted decay length and the given ambipolar diffusion length with varied excitation spot size. The *x* = *y* line is shown as the dashed line for clarity. (**e**) Fitted decay length as a function of the surface recombination velocity with varied carrier lifetime. (**f**) Fitted decay length as a function of the pumping density. The profiles in (**a**-**c**) are offset vertically for clarity. The exponential functions with different given *L*_*a*_ are shown as the dashed lines in (**a**-**c**) for comparison. The given ambipolar diffusion lengths are shown in (**e**,**f**) as the dashed lines for clarity. The pulse excitation conditions are the same as those in Fig. 1. *D*_*a*_ is 20.4 cm^2^/s, *τ* is 254 ps, *C* is 7 × 10^−30^ cm^6^/s, and *S* at *x* = 0 boundary between GaAs and air is 10^5^ cm/s unless otherwise specified.

After the fitting formula is developed, the effectiveness of the fitting method will be verified with the help of numerical simulation. Figure 2a-c shows the profiles of *δN*_*trunc*_ under pulse excitation with varied given ambipolar diffusion length, surface recombination velocity, and pumping density. The exponential functions with different given *L*_*a*_ are shown as the dashed lines in Fig. 2a–c for comparison. The relation between the fitted decay length *L*_*fit*_ using the fitting function and the given ambipolar diffusion length *L*_*a*_ is shown in the Fig. 2d. The *x* = *y* line is shown in the figure as the dashed line for clarity. In order to reflect the finite signal-to-noise ratio in SPLM measurement, the profile of the normalized *δN*_*trunc*_ is first plotted in logarithmic scale, and then with the exclusion of the vicinity of the boundary, the linear region with *δN*_*trunc*_ value above ~1% of the peak value of the total photocarrier number *δN* is chosen as the fitting range. It is found that the fitted decay length obtained using this fitting scheme is 710 nm with the given ambipolar diffusion length of 720 nm. Both are consistent with each other very well. However, the fitted decay length will gradually differ from the *x* = *y* line with decreasing *L*_*a*_. The relative error of the fitting remains below 10% for diffusion length down to about 300 nm without any correction for the excitation spot size of 1 μm. The results with excitation spot size of 500 nm is also shown in the figure. It is clear that with reduced excitation spot size, e.g. through adopting a pumping laser with shorter emission wavelength, a smaller *L*_*a*_ with sufficient accuracy can be obtained. A rule of thumb is that the excitation spot size should be smaller than the triple of *L*_*a*_ for diffusion length extraction with relative error smaller than 10%. Figure 2e shows the fitted decay length as a function of the surface recombination velocity of the GaAs/air interface. The given ambipolar diffusion lengths are shown in the figure as the dashed lines. It is found that for the case with lifetime of 254 ps, the fitted decay length is consistent with the given value when surface recombination velocity between GaAs and air is larger than 6 × 10^4^ cm/s. When surface recombination velocity is lower, carriers will accumulate near the boundary, and then the photocarrier distribution will deviate from the ideal distribution ${K}_{0}(r\text{'}/{L}_{a})$. This may lead to large inaccuracy for diffusion length extraction in structures with low surface recombination velocity. Surface recombination velocity can be increased purposely for extracting diffusion length in materials with low surface recombination velocity^31,32^. It is found that for materials with longer carrier lifetime, for example, 1 ns, diffusion length extraction with sufficient accuracy can be achieved for lower surface recombination velocity. It should be noted that ambipolar diffusion in a semi-infinite GaAs plane at position *x* > 0 is considered here due to the assumption of charge neutrality of photocarriers at any point of space and time^25^. Any condition that violates this assumption will cause the proposed fitting method invalid. For example, net charge may exist at *x* = 0 boundary if either electrons or holes exhibit a low surface recombination velocity at the boundary. That will cause carriers drift in addition to diffuse. Figure 2f shows the fitted decay length as a function of the pumping density. It is found that the fitted decay length gradually differs from the given ambipolar diffusion length with increasing pumping density. Auger recombination becomes non-negligible when extracting the ambipolar diffusion length under strong excitation, making the photocarrier distribution deviate from the ideal distribution ${K}_{0}(r\text{'}/{L}_{a})$, and finally results in the deviation of extraction results. Thus, the pumping density for diffusion length extraction should be chosen with caution to avoid the unwanted influence of Auger recombination.

**Figure 3:**
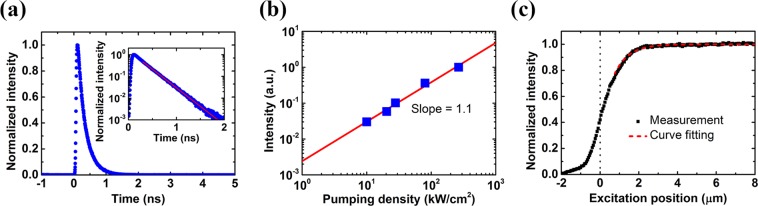
Experimental results of spatially-integrated PL in the GaAs thin film. (**a**) The measured temporal evolution of PL signal from the same sample far away from the edge. The logarithmic scale version of the temporal evolution is shown in the inset. (**b**) Log-log plot of the time-integrated PL intensity as a function of pumping density. The result of linear fitting is also shown in the figure, and the slope of the fitting line is 1.1. (**c**) Measured SPLM profile for the normalized time-integrated PL intensity. The fitting curve is also shown as the dashed red line for comparison. The dotted black line indicates the edge of the sample. The pumping density in (**a**,**c**) is about 290 kW/cm^2^.

After verification of the proposed fitting method, we apply this method to extract the ambipolar diffusion length in a GaAs thin film structure. The sample was grown by molecular beam epitaxy (MBE) on a (100) GaAs substrate. The sample structure is basically a 110 nm thick unintentionally doped GaAs layer capped by AlGaAs capping layers. See the Methods for details about the sample structure and preparation. Regarding the SPLM technique, conventional TRPL measurement setup is adopted with excitation position precisely controlled using piezo-electric manipulators. A mode-locked femtosecond Ti:sapphire laser was used for excitation. Detailed information about the measurement setup can be found in the Methods. Figure 3a shows the temporal evolution of the PL signal. The carrier lifetime is extracted to be 254 ps. The short carrier lifetime is also observed in epitaxial GaAs thin film using pump-probe setup in the literature^15^. The temporal evolution in logarithmic scale resembles a single-exponential function as shown in the inset of Fig. 3a, and this indicates that the Auger recombination is insignificant under such excitation condition. The single-exponential decayed intensity also justifies the monomolecular-recombination-dominant assumption during the development of the fitting method. Figure 3b shows the log-log plot of the time-integrated PL intensity as a function of pumping density. The result of linear fitting is also shown in the figure, and the slope of the fitting line is close to one. Together with the dominance of the monomolecular recombination process from Fig. 3a, we can conclude that the time-integrated PL intensity is proportional to the number of photocarriers. Figure 3c shows the measured SPLM profile for time-integrated PL intensity. The pumping density and the spot size were kept the same as those in the TRPL measurement in Fig. 3a. Excitation position is scanned across the edge of the epitaxial sample. Note that due to the finite spot size in the SPLM measurement, the signal in Fig. 3c is expected to extend outside the sample. In addition, for excitation position outside the sample, scattered excitation may also contribute to extra PL emission. According to Eq. (4), *L*_*a*_ can be extracted by subtracting the measured SPLM profile from its peak value and performing linear fitting in logarithmic scale. With the fitting method, the ambipolar diffusion length in the GaAs thin film is extracted to be 720 ± 30 nm which has been used in the simulation above. Therefore, the 2D transport model can be justified in this case since the thickness of the GaAs thin film is only 110 nm. A reference spatially-resolved PL measurement similar to the literature^8^ are performed for verification of the SPLM results, and the ambipolar diffusion length is determined to be about 700 nm. Therefore, the diffusion length extracted from SPLM is further verified by the spatially-resolved PL measurement. Detailed description of the spatially-resolved PL measurement is given in the Supplementary Information. With the carrier lifetime measured in Fig. 3a, ambipolar diffusion coefficient is obtained to be between 19.1 and 22.4 cm^2^/s. The extracted ambipolar diffusion coefficient in GaAs thin film is consistent with the value found in the literature using the pump-probe technique^15^.

## Conclusion

In conclusion, 2D ambipolar diffusion in semiconductor thin film structures was investigated using an SPLM technique. A new simple fitting method is proposed to extract the ambipolar diffusion length with excitation-position-dependent PL measurement across the edge of a semiconductor. The method was first verified using numerical simulation. The effectiveness of the fitting method with different ambipolar diffusion length, surface recombination velocity, and pumping density was discussed. The fitting method was then demonstrated to analyze the measured SPLM profile of a GaAs thin film structure. Carrier lifetime was extracted in an accompanying TRPL measurement under the same excitation condition, and thus the ambipolar diffusion coefficient can be determined simultaneously. With the new fitting method, we will be able to evaluate carrier transport properties in 2D electronic transport structures such as thin films or quantum wells.

## Methods

### Sample preparation

The sample was grown by molecular beam epitaxy (MBE) on a (100) GaAs substrate. A 1 μm thick Al_0.8_Ga_0.2_As layer followed by a 110 nm thick unintentionally doped GaAs layer with a 10 nm thick Al_0.35_Ga_0.65_As capping layer on top. The surface recombination velocity of the GaAs/AlGaAs interface is reported to be about tens to hundreds of cm/s^33,34^, which is much smaller than the surface recombination velocity of the GaAs/air interface. Therefore, the surface recombination at GaAs/AlGaAs interface is neglected in this study. It should be noted that the assumption of charge neutrality is imposed for SPLM measurement. Any condition that violates this assumption will cause the proposed fitting method invalid. Van der Pauw and Hall measurements showed typical resistivity of 0.1–0.2 Ω ∙ cm and n-type carrier concentration of 1–2 × 10^16^ cm^−3^. In order to avoid the emission contribution of the GaAs substrate during the PL measurement, the sample for measurement was mounted upside down onto a glass substrate followed by a GaAs substrate removal process using HNO_3_-based and C_6_H_8_O_7_-based solutions^35,36^. It should be noted that the edge of the GaAs layer must be free from the mounting material so that the optical signal can be correctly detected during SPLM measurement.

### Scanning photoluminescence microscopy (SPLM)

Conventional TRPL measurement setup is adopted. The excitation position was precisely controlled using piezo-electric manipulators. A mode-locked 776 nm femtosecond Ti:sapphire laser with pulse width of 130 fs and repetition rate of 76 MHz was used for excitation. The pump laser was focused by a 100X objective lens, and the spot size is 1 μm. The pumping density is about 290 kW/cm^2^. Discussion about pumping conditions and resulted carrier densities in simulation and the experiment is given in the Supplementary Information. A single-photon avalanche photodiode and a photon counter were used to detect the emission of the sample. Spatially-integrated PL intensity is then recorded with scanning excitation position. Note that the field of view of the focusing objective lens is larger than 120 μm, which is limited by other optical elements in the optical setup. Considering the extracted diffusion length of 720 nm, the spatial range that contributes to 99% of the PL intensity is less than 7 μm. Within such a small range, there is no vignetting experimentally observed. Therefore, the PL emission of the GaAs sample can be unbiasedly collected by our setup. Compared with most carrier diffusion length measurement using photoluminescence imaging, no charge-coupled device (CCD) or CMOS array is needed in our optical setup.

## Supplementary information

Supplementary Information.

## Data Availability

The data reported in this paper are available upon request.
