# Supplementary material for: A New Fitting Method for Ambipolar Diffusion Length Extraction in Thin Film Structures Using Photoluminescence Measurement with Scanning Excitation
Source: Sci Rep. 2020 Mar 23;10:5200. doi: 10.1038/s41598-020-62093-w (PMC7090066; doi:10.1038/s41598-020-62093-w)
Supplement: Supplementary file 1 — Supplementary Information. [file 41598_2020_62093_MOESM1_ESM.pdf]

Supplementary Information

A New Fitting Method for Ambipolar Diffusion Length Extraction in Thin Film Structures Using Photoluminescence Measurement with Scanning Excitation

Cheng-Hao Chu,[1] Ming-Hua Mao,[1,2,3,*] You-Ru Lin,[3] and Hao-Hsiung Lin[1,2,3]

[1]Graduate Institute of Electronics Engineering, National Taiwan University, No. 1, Roosevelt Rd. Sec. 4, Taipei 10617, Taiwan

[2]Department of Electrical Engineering, National Taiwan University, No. 1, Roosevelt Rd. Sec. 4, Taipei 10617, Taiwan

[3]Graduate Institute of Photonics and Optoelectronics, National Taiwan University, No. 1, Roosevelt Rd. Sec. 4, Taipei 10617, Taiwan

*Correspondence and requests for materials should be addressed to M.-H.M. (email: mhmao@ntu.edu.tw)

Development of the analytic formula for scanning photoluminescence microscopy (SPLM). It is found that with an excitation source located near a recombination boundary at $x = 0$, the photocarrier distributions remain almost the same except being truncated at the boundary. Therefore, the total photocarrier number δN with a given excitation position x_{pump} can be approximated as the total photocarrier number in an infinite plane minus the truncated photocarrier number which can be calculated from the integral of the ideal photocarrier distribution in an infinite plane δn_{inf} in the truncated source free region. The expression of δN may be written as

$$\begin{aligned}\delta N(x_{pump}) &\equiv d\int_{-\infty}^{\infty}\int_{0}^{\infty}\delta n(x-x_{pump},y)dx\,dy\\ &\approx d\int_{-\infty}^{\infty}\int_{-\infty}^{\infty}\delta n_{inf}(x-x_{pump},y)dx\,dy - d\int_{-\infty}^{\infty}\int_{-\infty}^{0}\delta n_{inf}(x-x_{pump},y)dx\,dy\\ &= d\int_{0}^{2\pi}\int_{0}^{\infty}\delta n_{inf}(r)rdr\,d\theta - d\int_{-\infty}^{\infty}\int_{-\infty}^{0}\delta n_{inf}(x-x_{pump},y)dx\,dy\\ &\equiv \delta N_{inf} - \delta N_{trunc}(x_{pump})\end{aligned} \quad \text{(S1)}$$

where δn is the photocarrier concentration in the transport plane, d is the thickness of the transport plane, δN_{inf} is the total photocarrier number in an infinite plane, and δN_{trunc} is the truncated photocarrier number for excitation at $x = x_{pump}$.

Relation between photocarrier distributions under pulse excitation or continuous-wave (CW) excitation. For a linear time-invariant system, the unit step response $a(t)$ is given by[1,2]

$$a(t) = h(t) * u(t) = \int_{-\infty}^{\infty} h(t-\tau)u(\tau)d\tau = \int_{-\infty}^{\infty} h(\tau)u(t-\tau)d\tau = \int_{-\infty}^{t} h(\tau)d\tau = \int_{0}^{t} h(\tau)d\tau \quad \text{(S2)}$$

where $u(t)$ is the unit step function and $h(t)$ is the impulse response. The unit step response can be expressed as the time integral of the impulse response. For $t \to \infty$, the unit step response will reach its steady state value. Based on the concept, the time-integrated photocarrier distribution under pulse excitation δn can be written as

$$\delta n(x,y) = \int_0^\infty \delta n'(x,y,t)dt \propto \delta n_{CW}(x,y) \quad \text{(S3)}$$

where $\delta n'$ is the instantaneous photocarrier distribution per unit time under pulse excitation and δn_{CW} is the photocarrier distribution under CW excitation. The above expression shows that time-integrated photocarrier distribution under pulse excitation, which can be represented by the time integral of the impulse response with $t \to \infty$, is actually proportional to photocarrier distribution under CW excitation, which is the unit step response with $t \to \infty$. Therefore, the time-integrated photocarrier distribution under pulse excitation equals the photocarrier distribution under CW excitation, and both have the form of the steady state solution $K_0(r'/L_a)$ of the ambipolar diffusion equation outside the source region.

Spatially-resolved photoluminescence (PL) measurement. A spatially-resolved PL measurement similar to the literature[3] was performed for the verification of SPLM results. The GaAs sample was pumped using a 532 nm continuous-wave (CW) laser and a 100X objective lens. The excitation spot size is measured to be 1.7 μm. The spatially-resolved PL emission was collected using a high resolution camera. Since the excitation spot size is in the same order of magnitude with the expected ambipolar diffusion length, numerical simulation was then applied to fit the experimental data, and the ambipolar diffusion length is determined to be about 700 nm. Therefore, the diffusion length extracted from SPLM is further verified by the spatially-resolved PL measurement.

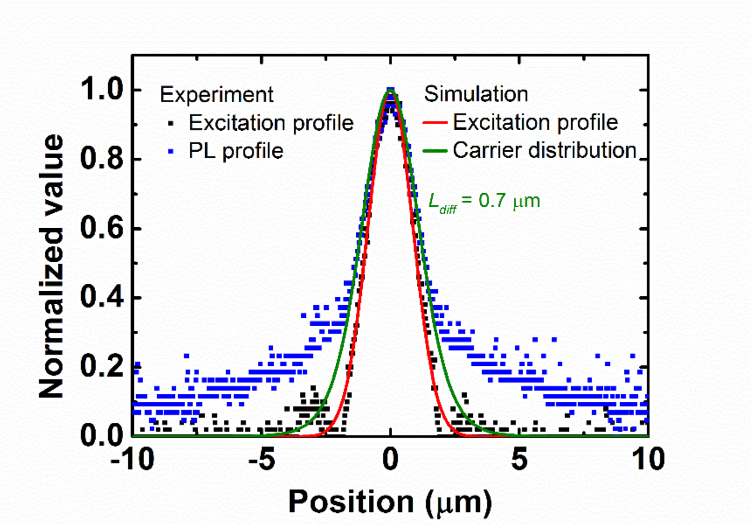

Figure S1. A spatially-resolved PL measurement in the GaAs thin film. The excitation spot size is measured to be 1.7 μm. The results using numerical simulation are also shown in the figure.

Discussion about pumping conditions and resulted carrier densities in simulation and the experiment. From the pumping density 290 kW/cm^2 used in the SPLM experiment and absorption coefficient of GaAs, carrier generation rate can be calculated and then be put into the simulation program to obtain the total photocarrier number δN as a function of time shown in Fig. S2. Simulation with other pumping densities is also included for comparison. With pumping density higher than 290 kW/cm^2, the time evolution of total photocarrier number begins to deviate from the single exponential decay due to Auger recombination. From the TRPL experiment, spatially-integrated PL shows single exponential decay with time. Therefore, the actual carrier density may be somewhat lower than that in simulation with pumping density 290 kW/cm^2. According to Fig. 2f, the proposed fitting method for SPLM is eligible for lower carrier density case due to lower

pumping density. Therefore, the proposed fitting method for SPLM can be appropriately applied in our experiment.

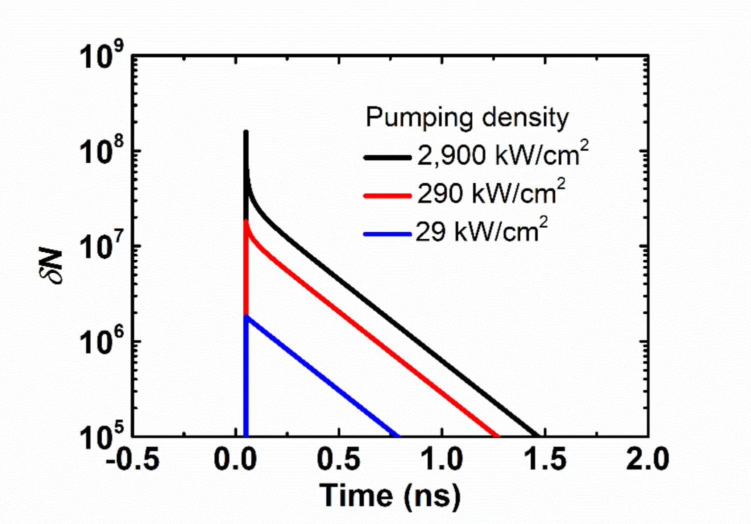

Figure S2. Time evolution for total photocarrier number δN with varied pumping density.

Influence of heating effect from excitation source on the ambipolar diffusion length extraction. Numerical simulation with heating effect was performed for GaAs under fs excitation. The diffusion length coefficient as a function of temperature in the simulation was calculated from the relation proposed by Blakemore.[4] Due to the heating of the excitation, the diffusion coefficient decreases non-uniformly in space as shown in Fig. S3a. For example, a 30K temperature increase from room temperature will lead to about a 10% drop of the ambipolar diffusion coefficient. However, it is found in Fig. S3b that the variation of the diffusion coefficient in space have only minor influence on the carrier distribution.

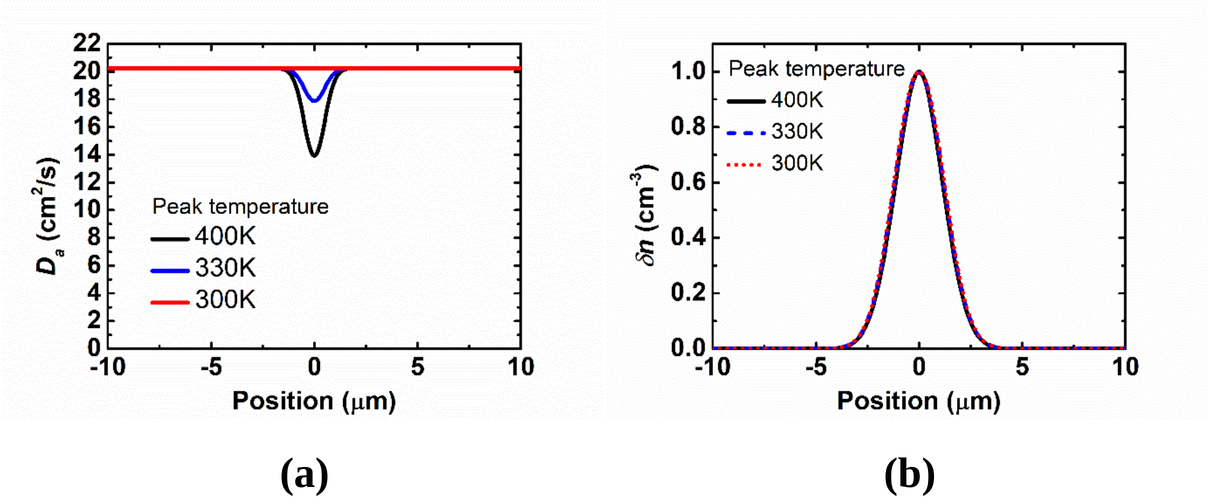

Figure S3. Numerical simulation with heating effect from pulse excitation source. (a) The spatial variation of ambipolar diffusion length due to heating from excitation source with varied peak temperature. (b) Normalized time-integrated photocarrier distribution with varied peak temperature.

References

1. Boyce, W. E. & DiPrima, R. C. *Elementary Differential Equations and Boundary Value Problems*. 346-354 (John Wiley & Sons, 2005).
2. Oppenheim, A. V., Willsky, A. S. & Nawab, S. H. *Signals and Systems*. 103-115 (Prentice Hall, 1997).
3. Paget, D. *et al.* Imaging ambipolar diffusion of photocarriers in GaAs thin films. *J. Appl. Phys.* **111**, 123720 (2012).
4. Blakemore, J. S. Semiconducting and other major properties of gallium arsenide. *J. Appl. Phys.* **53**, R123-R181 (1982).
